# Shuganyin decoction improves the intestinal barrier function in a rat model of irritable bowel syndrome induced by water-avoidance stress

**DOI:** 10.1186/s13020-017-0161-x

**Published:** 2018-02-01

**Authors:** Lu Lu, Liang Yan, Jianye Yuan, Qing Ye, Jiang Lin

**Affiliations:** 1 Department of Gastroenterology, Longhua Hospital Affiliated to Shanghai University of Traditional Chinese Medicine, Shanghai, 200032 China; 2 Department of General Surgery, Shuguang Hospital Affiliated to Shanghai University of Traditional Chinese Medicine, Shanghai, 201203 China; 3 Research Institute of the Spleen and Stomach Disease, Longhua Hospital Affiliated to Shanghai University of Traditional Chinese Medicine, Shanghai, 200032 China; 4 Department of Neurology, Longhua Hospital Affiliated to Shanghai University of Traditional Chinese Medicine, Shanghai, 200032 China

**Keywords:** Shuganyin decoction, Water-avoidance stress, Intestinal barrier function, Mast cell, Protease activated preceptor-2, Rat model

## Abstract

**Background:**

To determine the effect of Shuganyin decoction (SGD) on the intestinal barrier function in an irritable bowel syndrome (IBS) rat model induced by water-avoidance stress.

**Methods:**

Forty male Wistar rats were divided into control, water-avoidance stress (WAS) group, WAS plus Shuganyin decoction (SGD) group and WAS plus dicetel (Dicetel) group. IBS was induced in rats by subjecting them to water-avoidance stress for 7 days. On day 4 of the WAS protocol, the rats were treated for 7 consecutive days (days 4–11) with SGD, dicetel or a negative control (saline). The number of feces granules, histopathological changes of the intestine and mast cell (MC) morphometry were determined. Intestinal permeability was approximated by measuring the absorption of FITC-dextran 4400 (FD-4) from the lumen into the bloodstream in vivo and in vitro experiments. Also, the expression of protease active receptor-2 (PAR-2) and tumor necrosis factor-α (TNF-α) was estimated using immunohistochemical staining and ELISA, respectively. Tight junction (TJ) protein abundance was measured following a quantitative immunofluorescent analysis of intestinal sections and western blotting.

**Results:**

In vivo, WAS elicited a significantly increase in the transfer of FD-4 from the intestine to blood about threefold in 30 min compared with control group. After treated with SGD, the intestinal permeability to FD-4 of WAS-induced rats was significantly attenuated (*P* < 0.05). In vitro, the permeability coefficient (Papp) values were measured for FD-4 absorption across the excised intestine. WAS was shown to increase the intestinal permeability to (4.695 ± 0.3629) × 10^−7^ cm/s in 120 min, which was 2.6-fold higher than the control group. Rats treated with SGD showed a significant decrease in Papp values of FD-4 as compared to WAS group (*P* < 0.05). Furthermore, by immunofluorescent detection we found that WAS elicited the irregular distribution of TJ proteins. Using the quantitative analysis software of the medical image, the average optical density and protein abundance of TJ proteins was shown to be lower in the WAS group as compared to control group, (*P* < 0.05). SGD could attenuate this response and improve TJ distribution (*P* < 0.05). Western blot analysis confirmed that TJ protein abundance was significantly decreased in WAS group and that they could be returned to control levels following an SGD treatment. WAS also induced an increase in number of MCs, their area and diameter as compared to controls. These observations were attenuated with an SGD or dicetel treatment. Similarly, the expression of PAR-2 and TNF-α exceeded control values in the WAS group and were shown to be successfully attenuated with an SGD treatment.

**Conclusion:**

WAS-induced IBS rat model exhibited intestinal barrier dysfunction, which was manifested as tight junction damage and structural rearrangements that increased the intestinal permeability. Under these conditions, MCs were activated and degranulated in the intestinal mucosa leading to the activation of PAR-2. Our data showed that SGD could inhibit the activation of MCs and down-regulate the expression of both PAR-2 and TNF-α. In turn, this was shown to improve the expression and structural arrangement of TJ proteins in the intestinal mucosa, thereby regulating the intestinal permeability. It was concluded that Shuganyin could protect the intestinal barrier.

**Electronic supplementary material:**

The online version of this article (10.1186/s13020-017-0161-x) contains supplementary material, which is available to authorized users.

## Background

Irritable bowel syndrome (IBS) refers to a commonly seen functional bowel disorder with the features of chronic abdominal pain or discomfort and the change in bowel habits. IBS has a major influence on patients’ life quality. In the past, most reports in the literature focused on the study of gastrointestinal motility disorders and visceral hypersensitivity. Motility disorders and hypersensitivity are the main pathophysiologic consequences of IBS [1, 2]. However, more recent studies show the innerconnection between intestinal barrier dysfunction and immune activation [3, 4]. Furthermore, according to existing data, IBS patients with predominant diarrhea have a higher degree of intestinal permeability [5–7]. The pathophysiological signals leading to the destruction of intestinal permeability in IBS are still unclear.

Mast cells (MCs) are important regulators of the immune response, especially in the gastro-intestinal tract. When pressure signals are conveyed through the brain–gut axis, MCs release proinflammatory mediators, including tryptase. In turn, these mediators stimulate nerve terminals and influence intestinal motility. Specifically, following their interaction with PAR-2 receptors on the epithelial cells, the TNF-α expression is increased and tight junction (TJ) proteins are modulated [8–11]. Ultimately, stress-induced changes to the brain–gut axis can result in intestinal hyperpermeability.

It is common knowledge that there is no standard therapeutic agent to cure IBS. Currently, the most frequently used therapeutic is dicetel. Dicetel is a calcium antagonist that acts as a gastrointestinal antispasmodic by inhibiting the calcium channels of the smooth muscle cells that line the intestinal wall, thus preventing excessive intestinal contraction. In addition, dicetel can block the expression of the nerve peptide, regulate the intestinal hypersensitivity and, thereby, improve abdominal pain and discomfort. Nevertheless, the therapeutic effects of dicetel have not been satisfactory. An alternative is Shuganyin, a traditional Chinese medicine that has gained some traction in the research community for the treatment of IBS. Although its underlying mode of action remains to be elucidated, Shuganyin has been successful for the treatment of IBS patients with disharmony of liver and spleen. Compared to dicetel, Shuganyin has the advantage of overall regulation.

The first aim of this study was to characterize the changes that can be observed in water-avoidance stress-induced rat model of diarrhoea-IBS by measuring the number and degranulation of MCs, the expression of PAR-2 and TNF-α proteins, the intestinal permeability, and the reorganization and abundance of TJ proteins. Secondly, we tested the hypothesis that Shuganyin can alleviate intestinal barrier dysfunction through a mechanism mediating the interaction between MCs, PAR-2, and the intestinal epithelium. The effect of Shuganyin on WAS-induced IBS in rats was determined by measuring the intestinal permeability, the spatial organization and the expression of TJ, and MC morphometry. The effects of Shuganyin were compared to a positive control treatment with dicetel, a calcium antagonist commonly prescribed to treat IBS.

## Methods

The Minimum Standards of Reporting Checklist contains details of the experimental design, and resources used in this study (Additional file 1).

### Preparation of Shuganyin decoction

SGD is comprised of the following five herbs: white atractylodes rhizome (Baizhu), 9 g; Paeoniae Radix Alba (Baishao), 6 g; Tangerine Peel (Chengpi), 6 g; Radix Saposhnikovia (Fangfeng), 4.5 g; and Radix Bupleuri (Chaihu), 6 g. And the total mass used was 31.5 g which is the common dose for adult humans. All the herbs were purchased from Yanghetang Pharmacy (Shanghai, China) as crude herbs. Dry powder of SGD’s aqueous extract was made by Herbal Chemistry Labomouseory of Shanghai University of Traditional Chinese Medicine. SGD were prepared as follows: firstly, 31.5 g crude SGD were made into powder and put in 8 cups of water for 12 h, boiled for at least 2 h, and filtered. Then, the liquid was collected. Repeat such procedures. Put the liquid from two steps together, dry them in vacuum until there is no aqueous phase. When they are dried in a vacuum drying oven, 125 g of SGD powder can be obtained separately. The crude drugs were extracted twice and the filtrates were combined and vacuum dried to obtain a solid aqueous extract. Each final gram contained 3.36 g of the initial crude drugs. The aqueous extract was prepared at Shanghai University of Traditional Chinese Medicine. Previous pharmacodynamics performed by our team found that the effect of middle dose SGD on IBS model is the best. So, we selected the middle dose SGD directly in this study [12].

### Animals water avoidance stress model

Male Wistar rats 250–280 g were purchased from Sino-British BK Lab Animal Ltd. (Shanghai, China) and acclimatized for 7 days after being delivered. All rats were allowed to get rodent chow and water and kept with 12 h light/dark cycle at 22 °C.

During the experiment, the rats were assigned to control (*n* = 10), WAS (*n* = 10), WAS plus dicetel (*n* = 10) (positive control), and WAS plus SGD (*n* = 10) groups. IBS was induced in rats subjecting them to water avoidance stress for 1 h/day for 10 days as previously described [11]. Briefly, rats were weighed and placed on a platform (8 × 6 cm) affixed to the center of a plastic container (55 × 50 cm) which is filled with water to 1 cm below the platform. On day 8, after rats were anaesthetized, sections of terminal ileum were wiped out instantly, for in vitro measurements. Ileum samples were dissected and stored at − 80 °C for histological analysis. In addition, another set of experimental rats (n = 10 per group) was used for in vivo intestinal permeability measurements.

### Animals drug treatment

On day 4 of the WAS protocol, rats were treated (*i.g.*) daily, 1 h before the stress for 7 consecutive days (days 4–10) with SGD, Dicetel or vehicle (saline). The conversion of the human dose to that used in rats was calculated based on body surface. Rats in the control and WAS groups were given saline (1.0 ml/200 g). Rats in the WAS plus SGD group were administered the Shuganyin decoction, that was prepared by mixing solid aqueous extract in distilled water (196.9 mg/ml), and administering a dose of 1.0 ml/200 g body weight. Rats in the Dicetel group received Pinaverium bromide (Pinaverium bromide, Lot Number: H20120127, Abbott Products SAS, Solvay Pharmaceuticals, France) that was ground into fine powder, dissolved in distilled water (4.2 mg/ml), and administered at a dose of 1.0 ml/200 g.

### In vivo measurement of intestinal permeability

Rats were anaesthetized with nembutal (45 mg/kg, i.p.) and cannulated via the jugular vein for blood collection. A midline abdominal incision was performed and the distal ileum was ligated and catheterized for administration of FD-4. Before closing the abdominal wall by suture, renal pedicles should be ligated. The intestinal mucosal permeability was evaluated by measuring the lumen-to-plasma entrance of FD-4. Tyrode’s solution (in mmol/l: NaCl 137, KCl 2.7, CaCl_2_ 1.8, MgCl_2_ 1.0, NaHCO_3_ 12, NaH_2_PO_4_ 0.4, glucose 5.5, PH 7.4) containing FD-4 (0.4 mg/ml) was injected into the intestinal (50 ml/kg), and blood samples (80 μl) were subsequently collected at 0, 15, 30, 60, 90 and 120 min. A fluorometer (485 nm excitation, 530 nm emission) was used to detect the FD-4 concentration in blood [13].

### In vitro mucosal permeability measurement

Rats were all anaesthetized and then euthanized, a section of distal ileum was immediately removed, washed, and then placed in oxygenated Krebs, then stripped of muscle layers gently. The mucosa layer was then mounted in a Ussing chamber (Physiological Instruments, San Diego, CA). The chamber opening exposed 0.5 cm^2^ of tissue surface area to 5 ml of circulating oxygenated Krebs buffer (in mmol/l: NaCl 107, KCl 4.5, NaHCO_3_ 25, NaH_2_PO_4_ 0.2, Na_2_HPO_4_ 1.8, CaCl_2_ 1.2, MgSO_4_ 1.0, glucose 12, PH 7.4) at 37 °C. Intestinal regions with visible damage were excluded from the studies. After the preparation had maintained stable for at least 30 min, FD-4 (0.1 mg/ml) was dropped in the mucosal reservoir. Samples (200 μl) were removed and supplied with identical volumes of fresh buffer at 0, 15, 30, 60, 90 and 120 min from the serosal side. The mucosal permeability was assessed by measuring transepithelial flux of FD-4, and was evaluated by apparent permeability (Papp) in cm/s as the equation: Papp = dQ/dt/AC. dQ/dt is FD-4 appeared in the membrane part. A is the tissue’s surface part, and C is the original compound concentration in the mucosal compartment [14].

### Immunohistochemistry

The intestines were washed out and tissue samples were kept in phosphate-buffered saline (PBS) containing 4% paraformaldehyde and 2 mM egtazic acid (EGTA). These tissue samples were then rinsed and agitated in PBS containing EGTA and sucrose before incubating them overnight at 4 °C. Tissues were embedded in optimum cutting temperature compound and cryostat sections (5 μm) were mounted on poly-l-lysine coated slides with acetone for 10 min at − 20 °C and rinsed in PBS-EGTA [15].

For the immunohistochemical staining of PAR-2, paraffin sections were dewaxed to water using standard procedures and the samples were rinsed three times with PBS. Then the tissue slides were incubated with the primary goat-anti-mouse PAR-2 antibody (Boster-Biological Technology, Wuhan, China) at 4 °C overnight. The tissues were incubated with second antibodies at room temperature for 20 min, after washed with PBS for five times. Following *Dolichos biflorus* agglutinin reaction, positive cells displayed brown–yellow particles in the cytoplasm. Digital images were collected from at least three random high power fields for further analysis.

For the immunohistochemical staining of TJ proteins ZO-1 and occludin, samples were labeled with either rabbit polyclonal C terminal anti-occludin antibody (rabbit polyclonal anti-ZO-1) or anti-occludin antibody raised against a 69 kDa fusion protein (Sigma, Shanghai, China) in a humidified chamber at 37 °C for 1 h. Samples were rinsed for three times with PBS and then embedded in PBS-glycerol. Approximately 20 tissue sections from each animal’s intestine sample were analyzed and imaged with a fluorescence microscope in a darkroom [16]. For F-actin immunofluorescence, samples were rinsed in PBS containing 0.5 μg/ml of TRITC-conjugated phalloidin for 30 min at room temperature. After washing with PBS with EGTA, the samples were evaluated and imaged with a fluorescence microscope in a blinded fashion.

Optical density values and masculine area were analyzed from digital images using the Medical Image Quality Analyze System (MIQAS) (Qiuwei Biotechnological Co. Ltd., Shanghai, China). IHC = positive area × optical density/total area.

### Histology

Full thickness segments of colon were fixed in paraformaldehyde, embedded in paraffin, and subsequently stained with toluidine blue (ECL, Beyotime, Nantong, China). Mast cells were counted at 400× magnification and non-overlapping areas above the muscularis mucosae were observated.

### ELISA

Intestine segments were washed repeatedly with a saline solution and the mucosal layer was scraped off. Homogenised mucosa was subsequently centrifuged to collect and store the supernatant at − 80 °C. TNF-α protein abundance was determined with an ELISA kit (JingMei Biotech, Beijing, China), according to the manufacturer’s protocol.

### Toxicity

After 7 consecutive days treatment with SGD, segments of liver, kidney, and lung tissues were fixed in paraformaldehyde and embedded in paraffin. Hematoxylin and eosin (H&E, Beyotime, Nantong, China) staining was used to observe the morphological changes in the liver, kidney and lung tissues at 200× magnification. Also, blood samples were taken from the abdominal aorta and centrifuged to obtain the serum. Levels of alanine aminotransferase (ALT), aspartate aminotransferase (AST), blood urea nitrogen (BUN), and creatinine (Cr) in the serum were estimated using biochemical kits (JingMei Biotech, Beijing, China).

### Western blot

Briefly, intestine segments were harvested and extracted with RIPA lysis buffer with a protease inhibitor cocktail (Sigma, Shanghai, China) for 30 min at 4 °C. Forty micrograms on each lysate were separated by 10% SDS-PAGE and conveyed to polyvinylidene difluoride (PVDF) membranes (Sigma, Shanghai, China). After overnight blocking with 5% nonfat dry milk, blots were sequentially incubated with primary (Sigma, Shanghai, China) and secondary antibodies (Sigma, Shanghai, China) for 60 min at room temperature. β acting was used as a loading control. Membranes were washed four times with TBST and incubated with anti-rabbit secondary antibodies (Sigma, Shanghai, China) for 2 h. Quantitative results were revealed by using the ECL detection system (GE Health-care).

### Statistical analysis

Results were expressed as the mean ± SD. One-way ANOVA was used for the determination of statistical significance as appropriate. For comparison of pathological scores, the Mann–Whitney rank sum test was used. *P* were considered as significance.

## Results

### SGD alleviated WAS-induced defecation

**Fig. 1:**
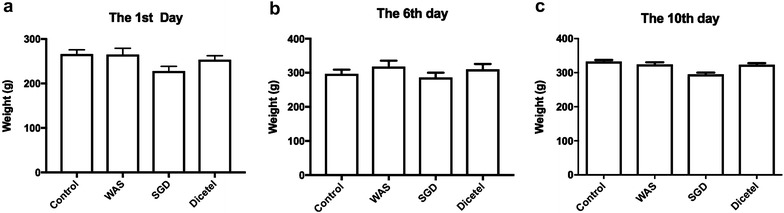
Body weights of control and WAS rats both before and after intervention with SGD or Dicetel. **a** Body weight of rats in the control, WAS, SGD and Dicetel groups on day 1 (before drug administration). **b** Body weight of rats in the control, WAS, SGD and Dicetel groups on day 6 (after drug treatment). **c** Body weight of rats in the control, WAS, SGD and Dicetel groups on day 10 (after drug treatment). Control, control group; WAS, water avoidance stress model group; SGD, WAS model + Shuganyin decoction; Dicetel, WAS model + Dicetel treatment as a positive control. n = 10 per group

During the WAS protocol, the body weight of the rats was measured before and after each stress period. The number of fecal granules was recorded within 1 h. Rats in the control group were weighed and the number of fecal granules was recorded in the same timeframe. No difference in body weight was observed between the groups on day 1 (*P* > 0.05) and on days 6 and 10 (respectively 3 and 7 days after administering SGC or dicetel) (Fig. 1).

**Fig. 2:**
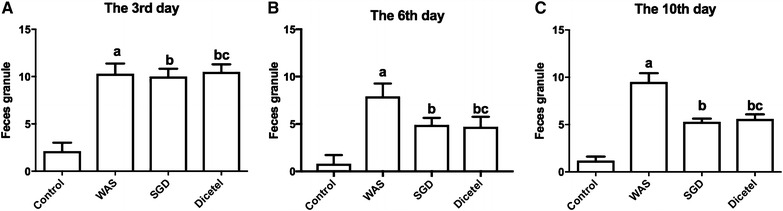
Control and WAS group defecation before and after treatment with SGD or Dicetel. **A** The number of feces from control, WAS, SGD and Dicetel groups recorded on day 3 (before drug treatment). **B** The number of feces from control, WAS, SGD and Dicetel groups recorded on day 6 (after drug treatment). **C** The number of feces from control, WAS, SGD and Dicetel groups recorded on day 10 (after drug treatment). Data were expressed as the mean ± SD. ^a^P < 0.01 compared to the control group; ^b^P < 0.01 compared to the WAS group; ^c^P > 0.05 compared to the SGD group. n = 10 per group

On day 3, prior to the administration of SGD or dicetel, the number of fecal granules collected from the WAS, SGD and Dicetel groups was increased above control levels (*P* < 0.01). Stool from the SGD and Dicetel groups appeared thin and soft. On days 6 and 10, at respectively 3 and 7 days after treatment with SGD or dicetel, the number of fecal granules for the SGD and Dicetel group rats was lower than for the WAS group (P < 0.01), but still higher than for controls (*P* < 0.01) (Fig. 2).

### SGD attenuated the WAS-induced increase in intestinal permeability

**Fig. 3:**
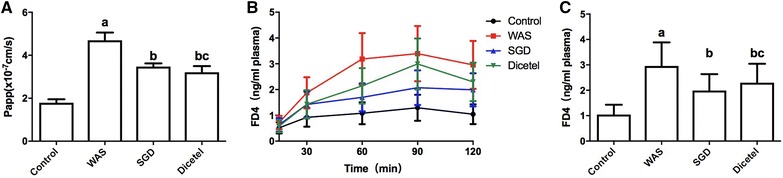
WAS-induced alterations in rat intestine permeability estimated by the rate of FD-4 transfer across the intestinal epithelium. **A**, **B** In vitro Papp values for FD-4 absorption across excised ileum. **C** In vivo detection of FD-4 in the blood stream 120 min after infusion into the lumen of the small intestine. Data were expressed as the mean ± SD. ^a^P < 0.05 compared to the control group; ^b^P < 0.05 compared to the WAS group; ^c^P > 0.05 compared to the SGD group. n = 10 per group

Changes in intestine permeability were determined by measuring FD-4 absorption through the intestinal epithelium in vitro and in vivo. In vitro, apparent permeability (Papp) values were estimated across excised segments of intestine. The ileum from rats subjected to WAS displayed a 2.6-fold increase in intestinal permeability (WAS group, 4.695 ± 0.363 × 10^−7^ cm/s in 120 min) above control Papp values. Rats treated with SGD showed a significantly lower FD-4 Papp value than the WAS group (Fig. 3A, B). In vivo, FD-4 absorption was estimated based on the amount absorbed into the bloodstream 120 min after an intraluminal injection of FD-4. Relative to controls, WAS caused an approximately threefold increase in FD-4 absorption across the intestinal mucosa. After treating with SGD, the intestinal permeability to FD-4 could be significantly attenuated. Moreover, the intestinal permeability of WAS-induced rats treated with dicetel was equivalent to SGD (Fig. 3C). Our in vivo data corroborated the aforementioned in vitro permeability estimates.

### WAS-induced ZO-1, occludin, and F-actin expression was reversed by SGD treatment

**Fig. 4:**
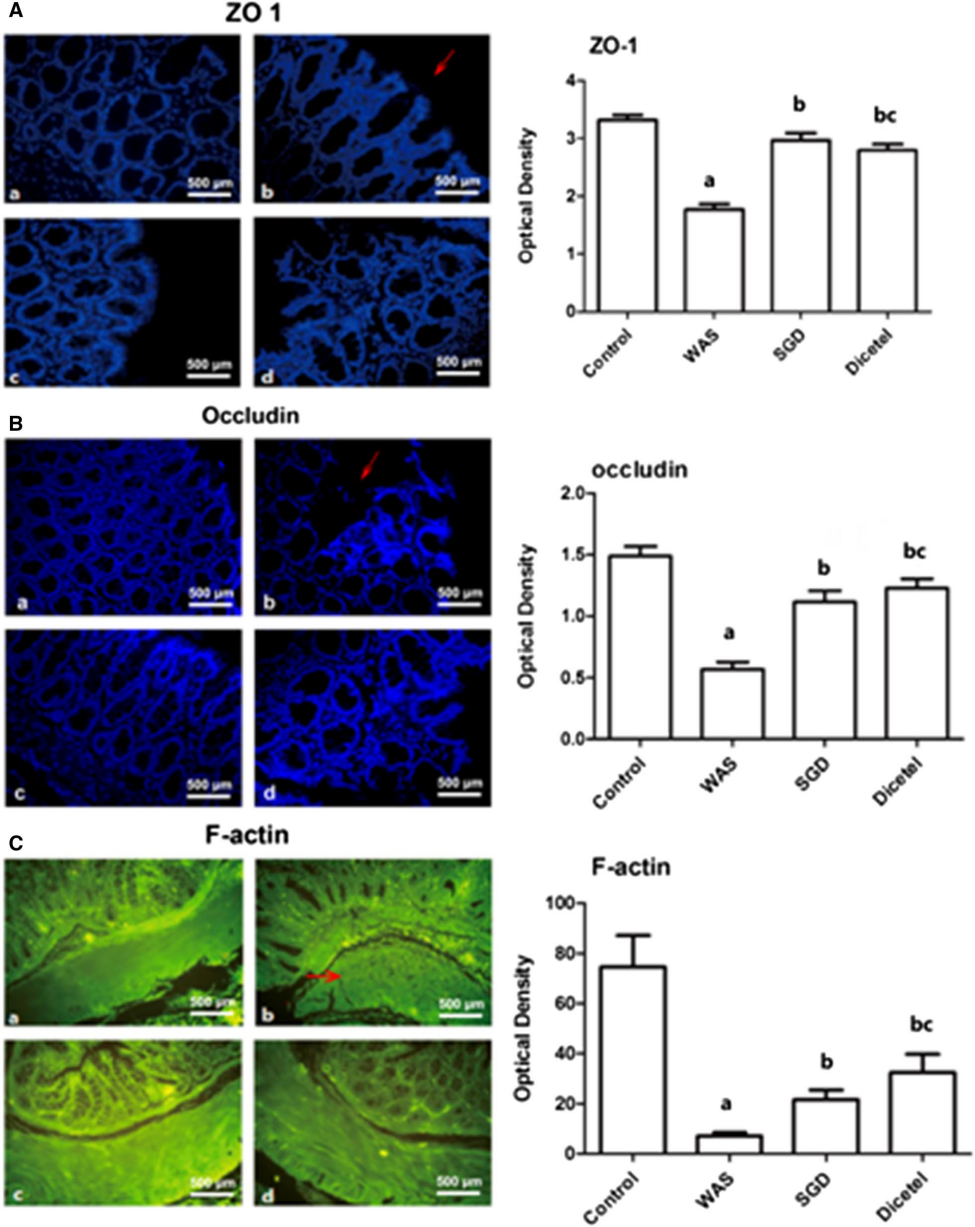
Immunohistological detection of ZO-1, occludin and F-actin protein levels in intestinal mucosa. **A** Immunohistological detection ZO-1 protein in the intestinal mucosa of animals from control group (**a**), WAS group (**b**), SGD group (**c**) and Dicetel group (**d**), (×400); average optical density levels of ZO-1 protein in the different groups (right panel). **B** Immunohistological detection of occludin protein levels in intestinal mucosa of animals from control group (**a**), WAS group (**b**), SGD group (**c**) and Dicetel group (**d**), (×400); average optical density levels of occludin in different groups (right panel). **C** Immunohistological detection of F-actin protein level in intestinal mucosa of animals from control group (**a**), WAS group (**b**), SGD group (**c**) and Dicetel group (**d**), (×400); average optical density levels of F-actin in different groups (right panel). Data were expressed as the mean ± SD. ^a^P < 0.05 compared to the control group; ^b^P < 0.05 compared to the WAS group; ^c^P > 0.05 compared to the SGD group. n = 10 per group

**Fig. 5:**
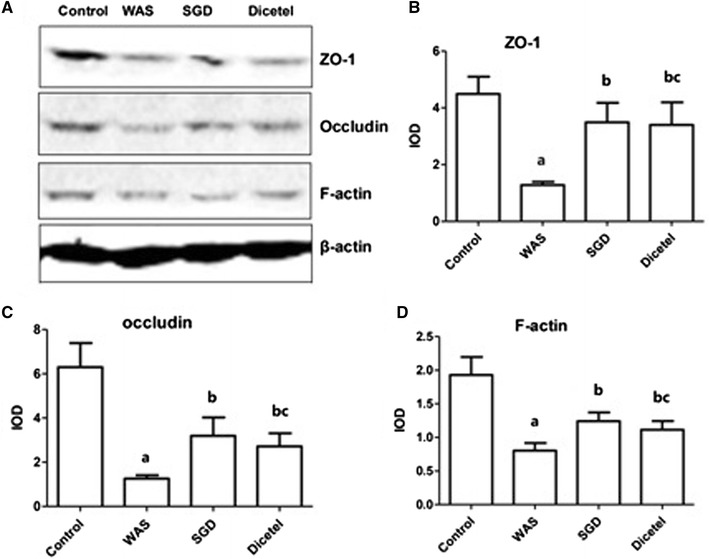
Western blot analysis of ZO-1, occludin and F-actin protein level in intestinal mucosa homogenates. **A** Western blot of ZO-1, occludin and F-actin proteins in intestinal mucosa homogenates from the control group, WAS group, SGD group and Dicetel group; **B**–**D** quantification the protein levels of ZO-1 (**B**), occludin (**C**) and F-actin (**D**) in the different experimental groups. ^a^P < 0.05 compared to the control group; ^b^P < 0.05 compared to the WAS group; ^c^P > 0.05 compared to the SGD group. n = 3–5 per group

As shown in Figs. 4 and 5, ZO-1, occludin and F-actin protein levels were significantly lower in the intestinal mucosa from the WAS rats than controls. Interestingly, WAS elicited an apparent disruption and irregular distribution of ZO-1, occludin and F-actin staining. Using image analysis software assay, the average optical density of each of these TJ proteins was found to be lower than in the control group. While a treatment with SGD attenuated the disrupted pattern of the three TJ proteins and the expression of ZO-1 and occludin, the levels of F-actin were only partially restored by SGD treatment. Similar observations were made for the positive control treatment with dicetel.

Western blot analysis showed that compared to the control group, protein abundance of the three TJ proteins was significantly decreased in the WAS group. Furthermore, SGD was shown to increase TJ protein abundance in WAS-induced rats.

### SGD decreased WAS-induced increases in mast cell number and size

**Fig. 6:**
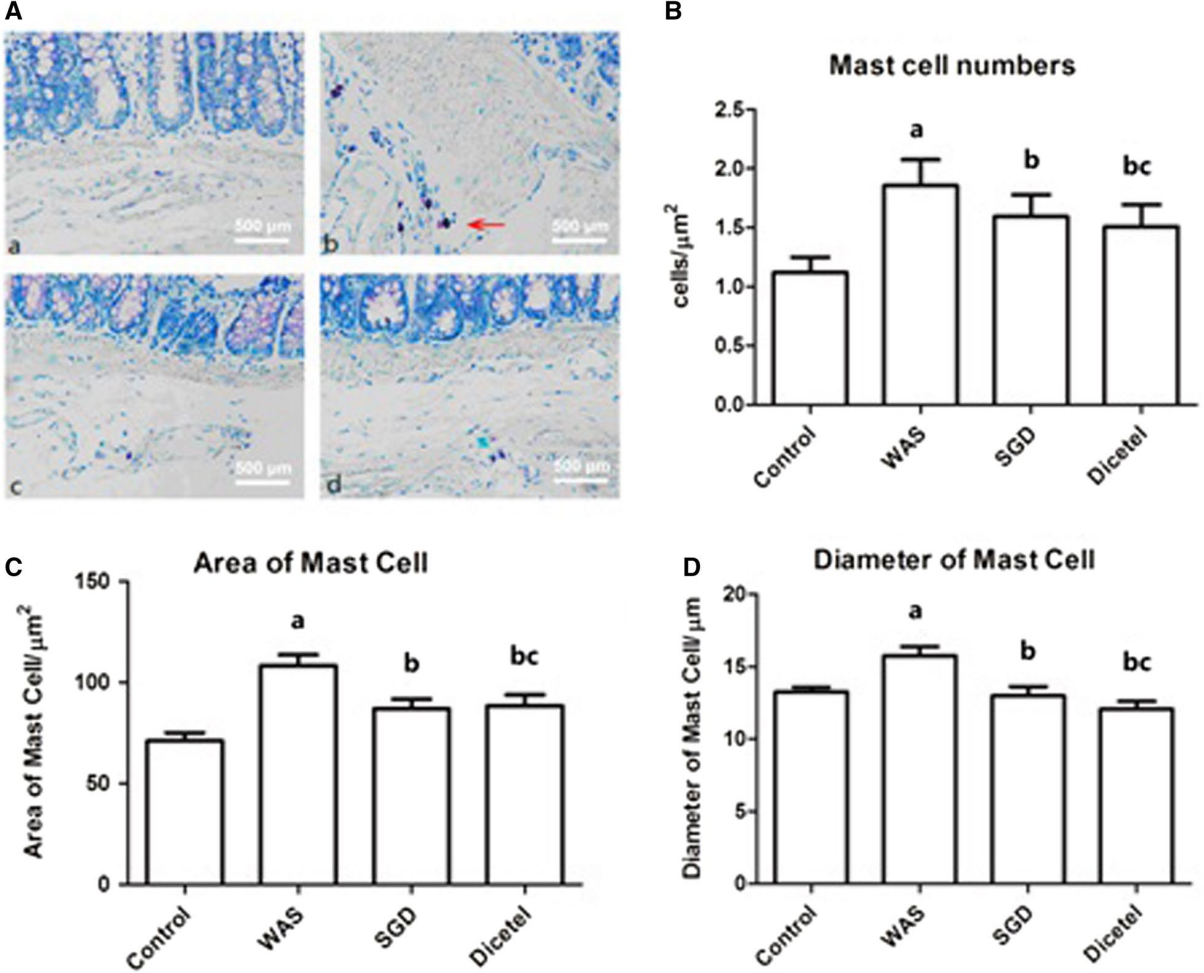
Morphology of mast cells in intestinal mucosa. **A** Toluidine blue staining revealing mast cells in the mucosa of animals from control group (**a**), WAS group (**b**), SGD group (**c**) and Dicetel group (**d**), (×200). **B**–**D** Quantification of mast cell characteristics in the mucosa and sub mucosa of colon. ^a^P < 0.05 compared to the control group; ^b^P < 0.05 compared to the WAS group; ^c^P > 0.05 compared to the SGD group. n = 10 per group

Microscopy of toluidine blue stained tissues revealed that the cytoplasm of MCs appeared granulated with coarse, violet–red pigments. Furthermore, the MCs were disseminated near small blood vessels in the mucosa and submucosa of the colon (Fig. 6A). MCs are characteristically round or spindle-shaped and have cytoplasmic membrane-bound granules. When the rats were subjected to WAS, MCs univocally appeared granulated, irregular in shape and unable to maintain plasma membrane integrity with granules leaking out of the cell. Treating these rats with SGD almost completely restored MC morphology. A similar observation was made when treating with dicetel. Also, the number, area and diameter of mast cells was calculated (Fig. 6B–D). The density, area, perimeter and diameter of the MCs were found to be significantly increased in the WAS group, compared to the control group. After treating with SGD or Dicetel, MC morphology returned to control appearance.

### SGD decreased WAS-induced activation of PAR-2

**Fig. 7:**
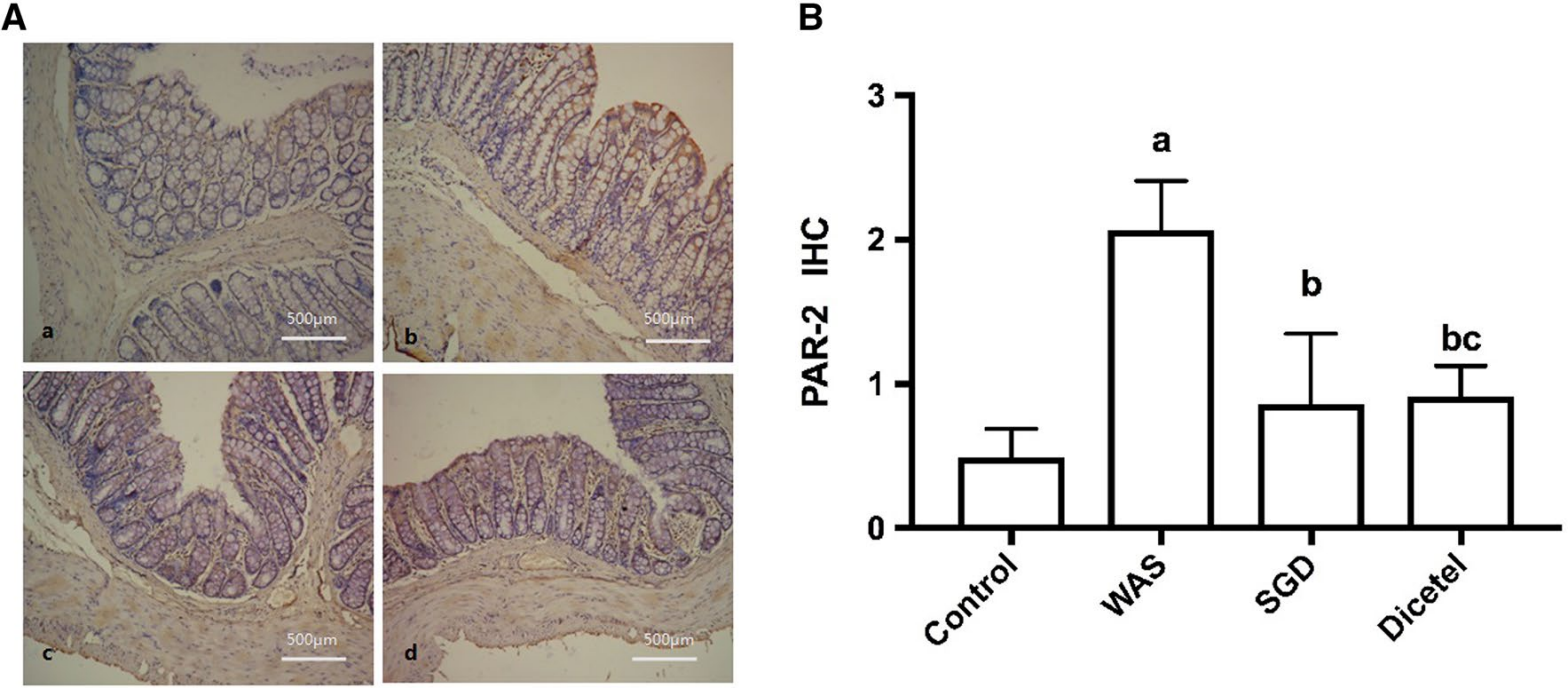
The immunohistochemical of PAR-2 in colon tissue. **A** Immunohistochemical staining revealing PAR-2 in the mucosa of rats from control group (**a**), WAS group (**b**), SGD group (**c**) and Dicetel group (**d**), (×200). **B** IHC of PAR-2 in different groups. Data were expressed as the mean ± SD. ^a^P < 0.05 compared to the control group; ^b^P < 0.05 compared to the WAS group; ^c^P > 0.05 compared to the SGD group. n = 10 per group

Based on the immunohistochemical staining, PAR-2 was expressed in the colon tissue and distributed in the cytoplasm. Using image analysis software, the average optical density of the PAR-2 staining was found to be higher in WAS-induced rats than in controls. SGD was able to significantly attenuate the expression of PAR-2 in WAS-induced rats as compared to the untreated WAS group (*P* *<* 0.05; Fig. 7).

### WAS-induced TNF-α expression was reversed by SGD treatment

**Fig. 8:**
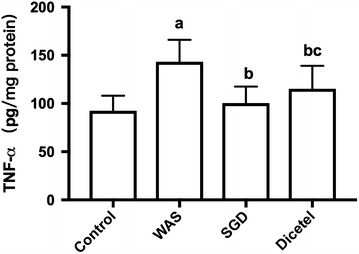
The expression of TNF-α in colon tissue. The expression of TNF-α in different groups. Data were expressed as the mean ± SD. ^a^P < 0.05 compared to the control group; ^b^P < 0.05 compared to the WAS group; ^c^P > 0.05 compared to the SGD group. n = 10 per group

Although H&E staining showed no inflammation in the colonic tissues, TNF-α abundance was higher in the WAS group than in the control group (*P* < 0.05). The previous was indicative of a low-grade mucosal inflammation in WAS-induced rat intestine. As for PAR-2, TNF-α expression in colonic tissue could be significantly reduced when treating the WAS-induced rats with SGD (*P* < 0.05; Fig. 8).

### The toxicity evaluation of SGD

**Fig. 9:**
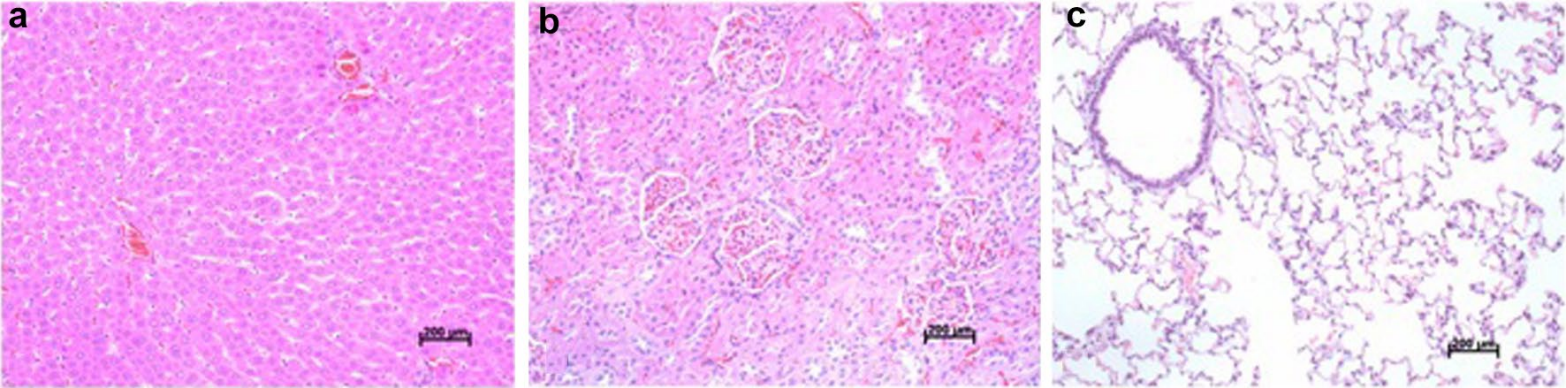
The morphological of liver, kidney and lung tissues stained with H&E after treated with SGD. H&E staining revealing in the liver tissue (**a**), kidney tissue (**b**), lung tissue (**c**) (×200)

**Fig. 10:**
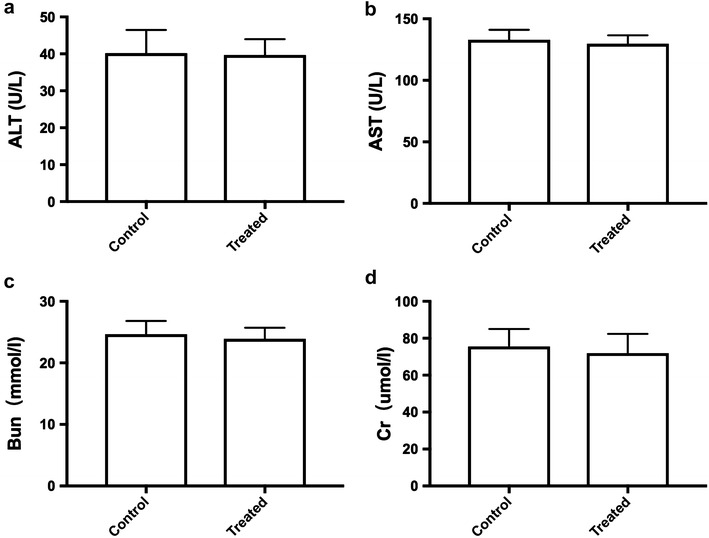
The biochemical parameters of rats after treated with SGD. **a** The expression of ALT before and after treated with SGD; **b** The expression of AST before and after treated with SGD; **c** The expression of BUN before and after treated with SGD; **d** The expression of Cr before and after treated with SGD. Data were expressed as the mean ± SD. n = 10 per group

Chinese medicine formulae may induce unexpected toxicity in vivo, we, therefore, examined liver, kidney and lung tissue slices and measured the serum levels of several biomarkers after 7 consecutive days of Shuganyin treatment. As shown in Figs. 9 and 10, there is no significant adverse event or changes in histopathological and biochemical parameters were recorded (*P* > 0.05).

## Discussion

IBS is usually considered as a disease of unknown cause, in addition to gastrointestinal motility disorders, visceral hypersensitivity, intestinal infections and brain–gut axis dysfunction, social background and psychological factors are considered to be closely related. Many reports have demonstrated that stress factors have a major impact on the epithelial cell permeability, however, whether psychological stress in patients with IBS would undermine the integrity of the intestinal barrier function remains unclear [17]. Currently, there are many views on the mechanism of intestinal barrier function changes under stress, including interaction between immune cells, intestinal neurons and epithelial cells mediated by corticotrophin-releasing hormone release, activation of the vagus nerve and mast cells secrete media [18–20].

Intestinal barrier function of intestinal epithelial means the inner partition intestine substance has the ability to prevent the pathogenic antigen intrusion. Intestinal barrier includes mechanical barrier, immunological barriers and bacteria barrier. Mechanical barrier is most important, which includes intestinal epithelial cells and the tight junction between cells. The so-called tight junction (TJ) locates between adjacent epithelial cells, which is the main connection between the intestinal epithelial cells, and plays a role in closing the gap cells within the intestine to prevent substance freely through the cell gap, through epithelial cell layer and regulating intestinal barrier permeability [21–23]. Tight junction contains a series of over 30 proteins [24], including blocking protein occludin and claudin, junction associated molecule (JAM) and other transmembrane proteins, cytosolic attachment protein ZO family (zonula occludens protein) and wire connected thereto actin-like protein. Among them, ZO-1 and occludin are most important. Filamentous actin F-actin, appears in all eukaryotic cells, and is an important component of the cytoskeleton. F-actin was associated with tight junction through a manner of patchy dense, they function together to regulate inner and outer cellular signal transduction pathways, change actin contractility, affect the assembly and function of tight junction, and regulate the permeability. Our study found that water-avoidance stress method almost destroyed all the tight junctions of intestinal mucosa. Western blot results showed that ZO-1, occludin, F-actin expressions were significantly reduced. In addition, the combination use of in vivo and in vitro method, showed that results of diarrhea-predominant IBS ileum permeability strongly suggest that water-avoidance stress model in rats can increased intestinal permeability. Thus, we assumed that the main cause of intestinal barrier dysfunction is the expression of tight junctions between epithelial cells reduced which leads to destruction of epithelial cells dense structure, and finally leads to increased intestinal permeability.

Studies have shown that the activation of immune cells in the intestinal mucosa is related to intestinal mucosal barrier disruption. The cytokine TNF-α is mainly secreted by monocytes and macrophages and associated with a range of physiological and pathological responses. TNF-α is known to induce apoptosis of intestinal epithelial cells and change the assembly of intercellular TJ proteins [25]. Martinez et al. found that, compared with healthy controls, intestinal mast cell activation expression of tryptase increased significantly, while the intestinal mucosa ZO-1 and protein ZO-3 decreased significantly [26, 27]. MCs are located in the blood vessels, nerves and mucous membranes nearby lymph and they act as the main antigen receptors regulating the immune response of the intestinal mucosa. When a specific antigen stimulates the immune system, sensory nerve endings excite and induce the production of IgE antibody. Following an antigen–antibody interaction MCs can activate, degranulate, and release a variety of biologically active mediators, of which tryptase is closely related to intestinal permeability. As a serine protease enzyme, tryptase is a strong protease-activated receptor activator of PAR-2. Activation of PAR-2 can induce a rearrangement of intestinal epithelial TJ and the cytoskeleton. This, in turn, changes the intestinal barrier structure and increases the permeability of the intestinal mucosa [28–30]. The study presented herein reported the changes inflicted by WAS-induced IBS in rats with measurements of including the intestinal mucosa PAR-2 expression, mast cells number, area, perimeter, roundness, and diameter. MCs were clearly activated because of the stress and some were even degranulating. The model was also used to test Shuganyin as a potential treatment of IBS and, indeed, following the treatment of WAS-induced rats with SGD, PAR-2 and intestinal TJ protein expressions were decreased to control levels. Also, the intestinal hyperpermeability due to the stress could be attenuated by SGD. Arguably, Shuganyin inhibits the activation and degranulation of mast cells. In turn, the release of biologically active mediators is reduced, inhibiting the rearrangement of intestinal epithelial TJs and thus reducing the permeability of the intestinal mucosa. Shuganyin is therefore believed to protect the intestinal barrier.

There were some limitations to the study as we did not use the PAR-2 high-pressure inhibitor GB88 [31]. A follow-up study using GB88 and an intestinal epithelial PAR-2 pressure specific gene knockout animal model has already been planned.

## Conclusions

The WAS-induced IBS rat model was shown to exhibit intestinal barrier dysfunction given the TJ damage and structural rearrangements that led to an increased intestinal permeability. Also, the activation and degranulation of MCs could be observed in their intestinal mucosa. When treated with Shuganyin, MC activation was inhibited and the expression of both PAR-2 and TNF-α was downregulated, whereas the expression of TJ proteins in the intestinal mucosa was increased. In addition, Shuganyin was shown to suppress the increase in intestinal permeability observed in rats subjected to WAS. It was concluded that Shuganyin can protect the intestinal barrier function.

## Additional file

**Additional file 1.** Minimum Standards of Reporting Checklist.

## Abbreviations

SGD: Shuganyin decoction
WAS: water-avoidance stress
PAR-2: protease active receptor-2
TNF-α: tumor necrosis factor-α
TJ: tight junction
FD-4: FITC-dextran 4400
IBS: irritable bowel syndrome
MC: mast cell
GI: gastro-intestinal
BGA: brain–gut axis
TCM: traditional Chinese medicine
Papp: apparent permeability
MIQAS: Medical Image Quality Analyze System
PVDF: polyvinylidene difluoride
